# Supplementary material for: Ocean temperature impact on ice shelf extent in the eastern Antarctic Peninsula
Source: Nat Commun. 2019 Jan 18;10:304. doi: 10.1038/s41467-018-08195-6 (PMC6338760; doi:10.1038/s41467-018-08195-6)
Supplement: Supplementary file 1 — Supplementary Information [file 41467_2018_8195_MOESM1_ESM.pdf]

# Supplementary information for:
# Ocean temperature impact on ice shelf extent in the eastern Antarctic Peninsula

Johan Etourneau[1,2], Giovanni Sgubin[1], Xavier Crosta[1], Didier Swingedouw[1], Verónica Willmott[3,4], Loïc Barbara[5], Marie-Noëlle Houssais[6], Stefan Schouten[3,7], Jaap S. Sinninghe Damsté[3,7], Hugues Goosse[9], Carlota Escutia[2], Julien Crespin[1], Guillaume Massé[8] & Jung-Hyun Kim[3,10]

[1]Instituto Andaluz de Ciencias de la Tierra, CSIC-Universidad Granada, Granada, Spain

[2]UMR 5805 EPOC, EPHE/CNRS, Université de Bordeaux, Pessac, France

[3]NIOZ Royal Netherlands Institute for Sea Research, and Utrecht University, Texel, The Netherlands

[4]Alfred Wegener Institute, Bremerhaven, Germany

[5]Instituto de Investigaciones Oceanologicas, Universidad Autónoma de Baja California, Baja California, Mexico

[6]UMR 7159 LOCEAN, CNRS/UPMC/MHNH/IRD, Université Pierre et Marie Curie, Paris, France

[7]Faculty of Geosciences, University of Utrecht, Utrecht, The Netherlands

[8]UMI CNRS Takuvik, Université de Laval, Québec, Canada

[9]Earth and Life Institute, Université de Louvain, Louvain-la-Neuve, Belgium

[10]Korea Polar Research Institute, Incheon, South Korea

**Water masses.** In comparison to the western part, permanent and automated weather stations are rare in the EAP as illustrated by the scarcity of small blue squares in Fig. S1. Moreover the oceanic system in the studied area is complex and poorly investigated. However, one study has been focused on local ocean currents at our coring sites[1].The authors found that the surface waters and bottom waters flowed eastward into the Prince Gustav Channel[1], which most likely results of seasonal sea-ice and ice sheet melting generating both cold and low salinity, cold and high salinity waters, respectively[1]. In contrast, subsurface waters (below 50m water depth) flow westward over the JPC-38 core site[1]. The source of this third water mass has not been characterized yet but is probably mainly originating in waters coming from the shelf and beyond. In order to assess the origin of this water mass through its hydrological characteristics, we used data generated during the austral summer at two nearby stations, one at the north and the other one south of the Prince Gustav Channel[2]. The two station profiles first clearly report a shift from which both temperature and salinity converge from 50 to 500m water depth towards similar values (-1°C and 34.5‰, respectively) (Fig. S1)[2]. Given the depth, this water mass cannot be associated with the Antarctic Surface Water (ASW) or remnant Winter Water (WW). Indeed, both latter water masses typically exhibit temperature and salinity range of -1.8 to -2ºC and 34.40 to 34.45‰, respectively, in the area[3]. They are mostly found in surface for the ASW and the first 200m for WW, especially when they flow offshore such as in the nearby Powell Basins3. In contrast to the bottom, the subsurface water at our study site cannot be influenced by the High Salinity Shelf Waters (HSSW), which flow from the ice shelf (e.g. Larsen A and B) towards the Weddell Sea before feeding the dense bottom waters[4]. In addition, the HSSW temperature is close to -2ºC and its salinity is around 34.6‰[3]. We also exclude a "pure" Warm Deep Water (WDW) signal, which cyclonically flows offshore through the Weddell Gyre or southward from the Bransfield eastern basin towards the northwestern part of the EAP (Gordon et al., 2000). Indeed, the WDW is characterized by positive temperature values (>0.5ºC) and higher salinity (34.65‰)[5]. Alternatively, we suggest that the subsurface water mass flowing through the Vega Drift corresponds to the modified version of the WDW (mWDW), which corresponds to a mixing between the WDW forced by westerlies-driven Ekman pumping and cold shelf waters (as illustrated on Figs.S1 and S3), similar to that previously detected along the northwestern Weddell Sea[5], the northern edge of the Larsen C ice shelf[6] or in Prydz Bay, East Antarctica[7]. This is consistent with previous studies from around Antarctica showing that this intermediate depth is typical of mCDW flow affecting the ice shelf base[8-9]. Because of its temperature above the seawater freezing point (-1.9°C, Fig. S1), the increasing WDW penetration into the continent when wind is getting stronger has the 38 potential to substantially erode the grounding line of the ice shelf throughout the Holocene and the recent decades and therefore control to a large extent its continuous retreat over the last 9,000 years.

**References**


1. Camerlenghi, A. et al. Glacial morphology and post-glacial contourites in northern Prince Gustav Channel (NW Weddell Sea, Antarctica). *Mar. Geophys. Res.* **22**, 417-443 (2001).
2. Olbers, D., Gouretski, V., Seiß, G. & Schröter, J. Hydrographic 147 Atlas of the Southern Ocean. Alfred Wegener Institute, Bremerhaven, 106 pages, ISBN (1992).
3. van Caspel, M., Schröder, M., Huhn, O. & Hellmer, H.H. Precursors of Antarctic Bottom Water formed on the continental shelf off Larsen Ice Shelf. *Deep-Sea Res. I* **99**, 1-9 (2015).
4. Thompson, A.F., Heywood, K., Schmidtko, S. & Stewart, A.L. Eddy transports as a key component of the Antarctic overturning circulation. *Nat. Geo.* **7**, 879-884 (2014).
5. Hellmer, H.H., Huhn, O., Gomis, D & Timmermann, R. On the freshening of the northwestern Weddell Sea continental shelf. *Ocean Sci.* **7**, 305-316 (2011).
6. Nicholls, K.W., Pudsey, C.J. & Morris, P. Summer time water masses off the northern Larsen C Ice Shelf, Antarctica. *Geophys. Res. Lett.* **31**, L09309 (2004).
7. Herraiz-Borreguero, L. et al. Circulation of modified Circumpolar Deep Water and basal beneath the Amery Ice Shelf, East Antarctica. *J. Geophy. Res.: Oceans* **120**, 3098-3112 (2015).
8. Jacobs, S. S., Hellmer, H., Doake, C.S.M., Jenkins, A. & Frolich, R. Melting of ice shelves and the mass balance of Antarctica. *J. Glaciol.* **38**, 375–387 (1992).
9. Joughin, I. & Padman, L. Melting and freezing beneath Filchner-Ronne Ice Shelf, Antarctica. *Geophys. Res. Lett.* **30**, doi:10.1029/2003GL016941 (2003).

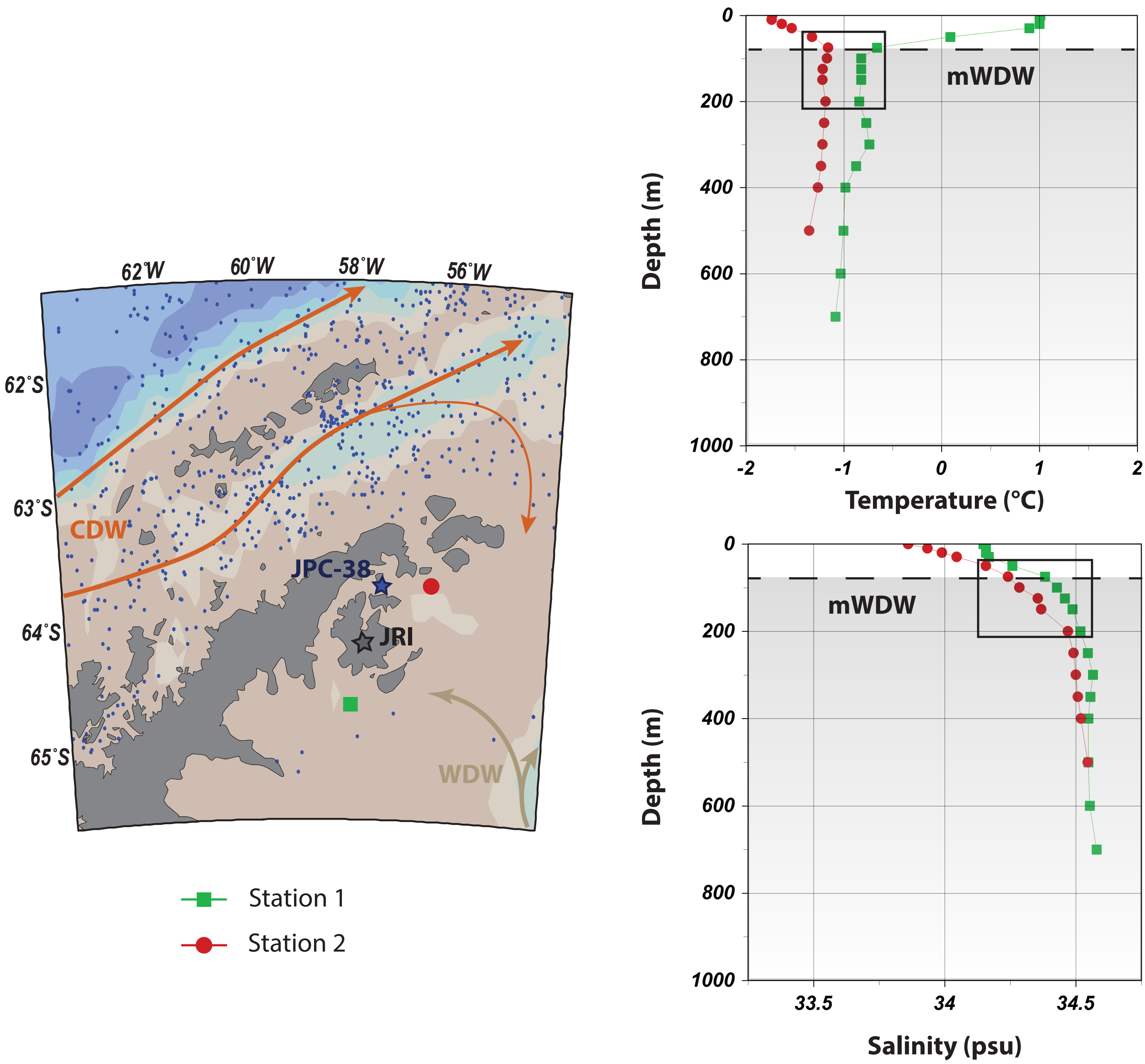


**Supplementary Figure 1.** (a) Location of the marine (black star) and ice core (white star) sites as well as the permanent or temporary stations (blue squares) where measurements have been collectedS2. The map includes the two stations (station 1 (green) and 2 (brown)) where the temperature (°C) and salinity (‰) profiles have been plotted in (b) and (c), respectively. The black arrow shows the circulation of the WDW along the coast and its penetration into the ice shelf.

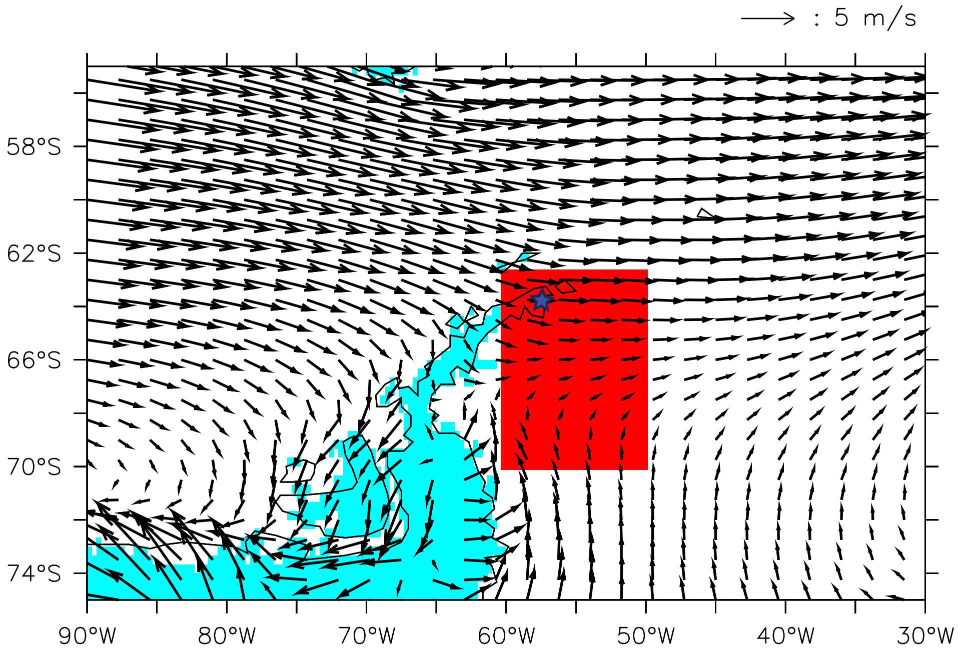


**Supplementary Figure 2.** In red is the area where the Ekman pumping has been computed using the NOAA-20CR8 and ERA-Interim version 2.016 reanalyses. Black arrows indicate the major wind direction and strength, i.e. the mean position of the southern westerly winds (SWW) favoring upwelling, and the southern winds, south of 66º30'S, conducive to mean downwelling conditions. The blue star corresponds to location of the site JPC-10.

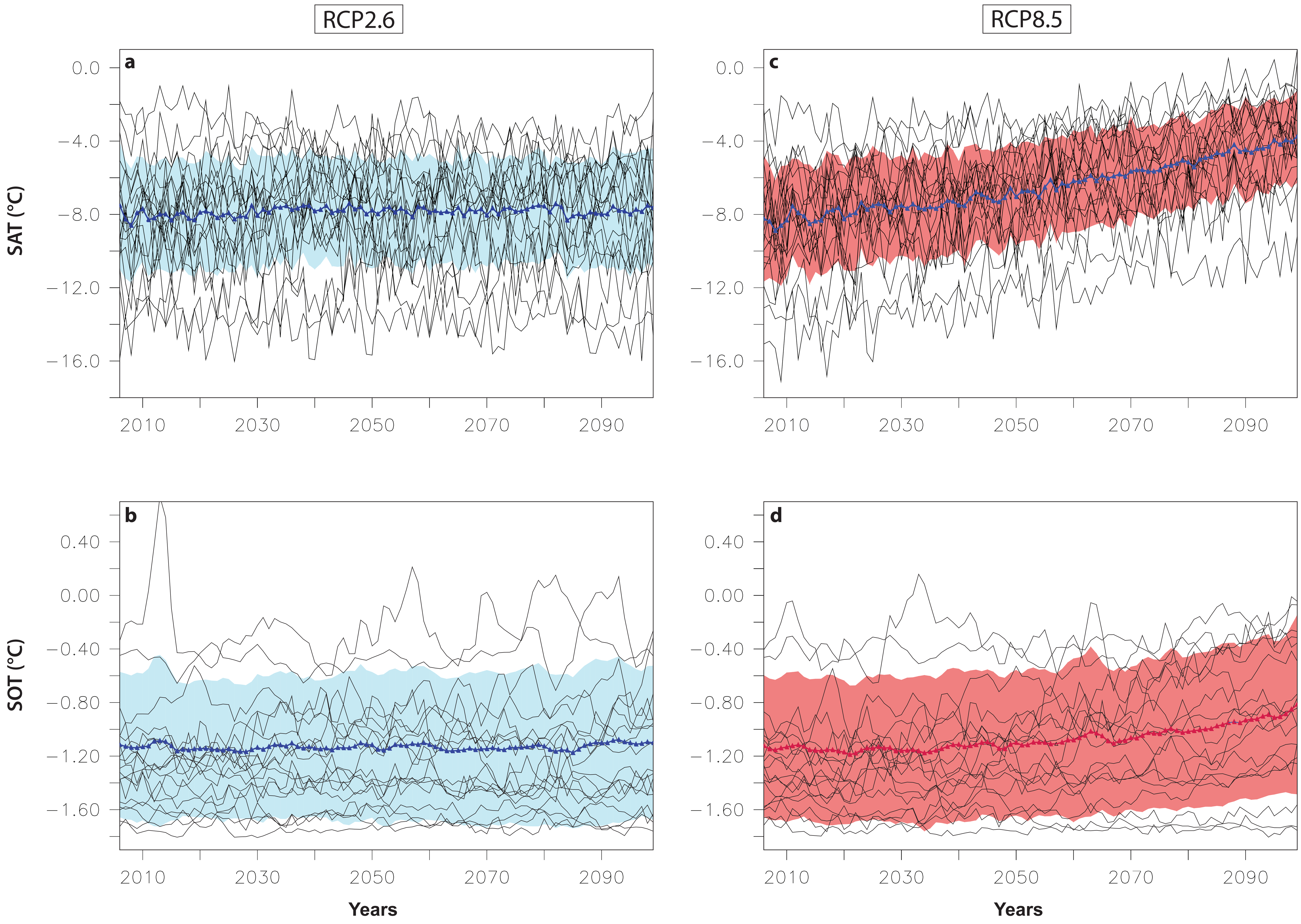


**Supplementary Figure 3.** The 26 projections from climate models showing the possible evolution of the SOT and SAT until 2100 using two different emission scenarios (RCPs): RCP2.6 and RCP8.5.

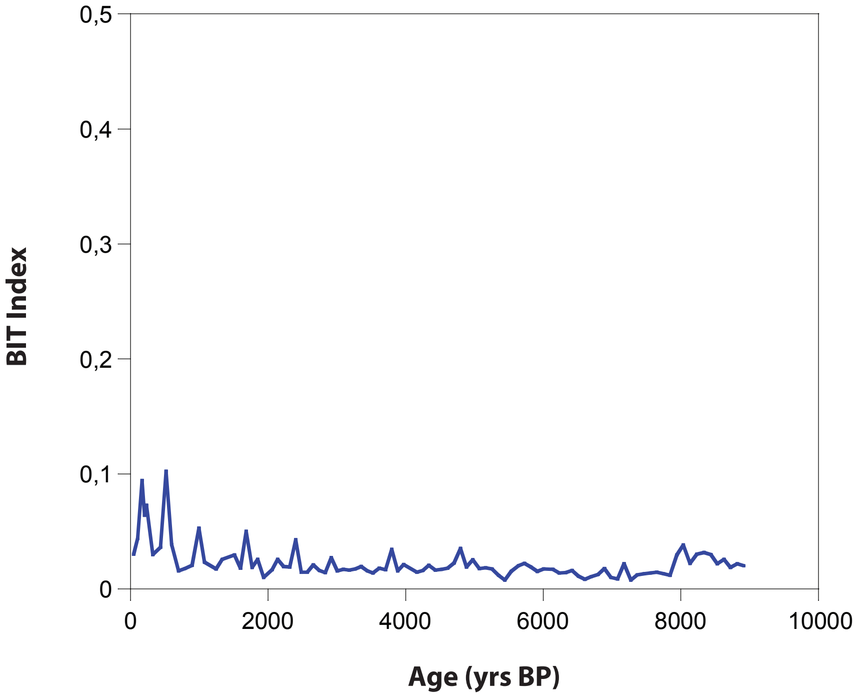


**Supplementary Figure 4.** BIT index calculated at the JPC-38 core site.

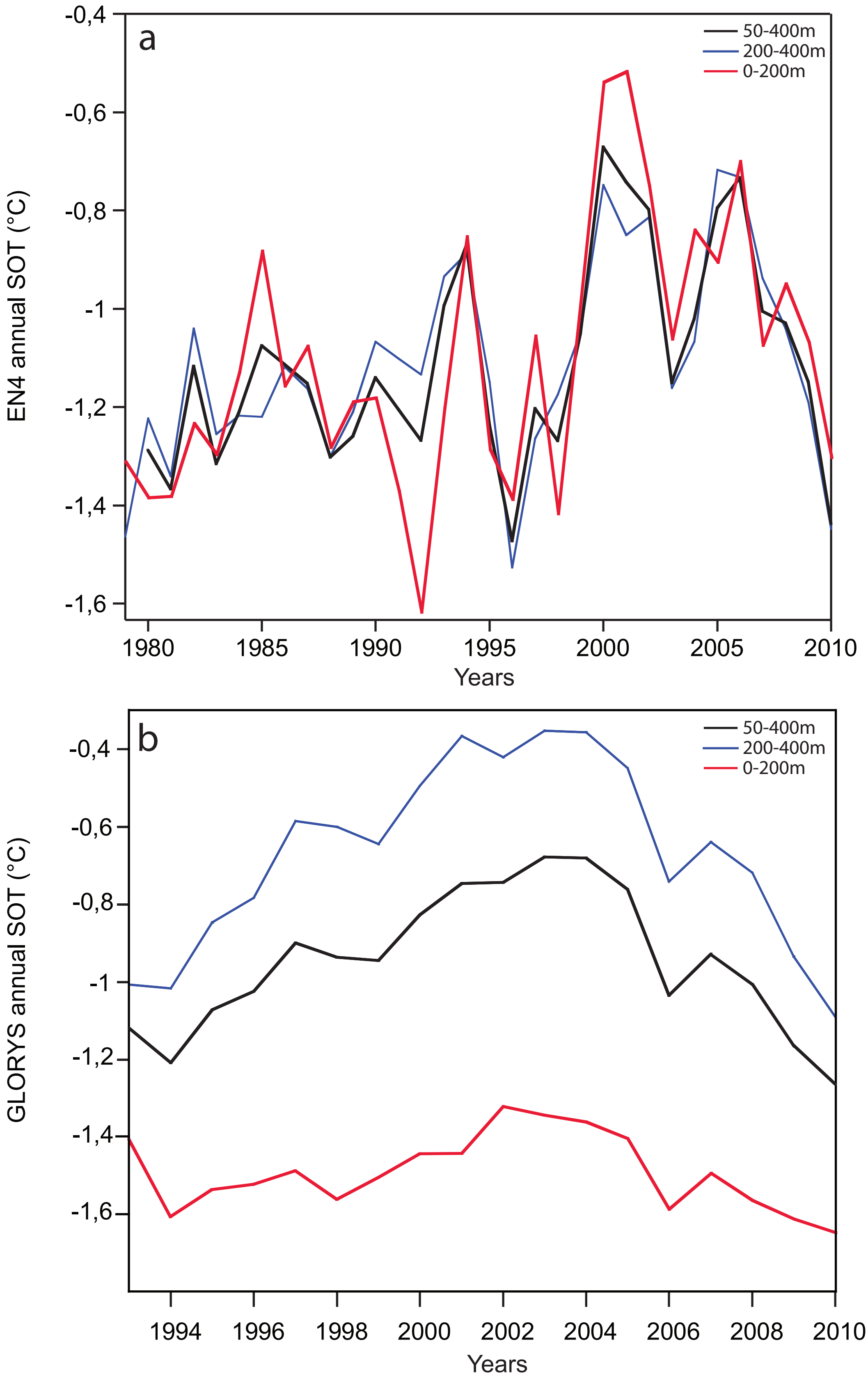


**Supplementary Figure 5.** (a) EN4 and (b) GLORYS temperature variations over the last decades at 0-200m, 200-400m and 50-400m water depths.
