# Supplementary material for: Ocean temperature impact on ice shelf extent in the eastern Antarctic Peninsula
Source: Nat Commun. 2019 Jan 18;10:304. doi: 10.1038/s41467-018-08195-6 (PMC6338760; doi:10.1038/s41467-018-08195-6)
Supplement: Supplementary file 3 — Description of Additional Supplementary Files [file 41467_2018_8195_MOESM3_ESM.pdf]

Description of Additional Supplementary Files

File Name: Supplementary Data 1
Description: Ferret code for Ekman pumping computation.
